# Mechanisms of bodily harm in emergency department youths with ADHD

**DOI:** 10.3389/frcha.2022.1033822

**Published:** 2022-10-17

**Authors:** Spencer I. Evans, Elijah W. Hale, Matt S. Silverman

**Affiliations:** ^1^School of Medicine, University of Colorado Anschutz Medical Campus, Aurora, CO, United States; ^2^Department of Anesthesiology, Yale New Haven Health System, New Haven, CT, United States

**Keywords:** ADHD, emergency care, trauma, bodily harm, pediatric emergency department

## Abstract

**Objectives:**

We sought to perform a review of emergency department data to illuminate whether there is a difference in the prevalence of severe injuries in patients with ADHD compared to patients without ADHD. We hope to illuminate whether providers should consider inquiring whether their pediatric patients have ADHD to improve long term outcomes.

**Methods:**

This study is a retrospective cohort study of patient records contained in the TriNetX database, specifically of pediatric patients in this database who presented to an emergency department. We specifically looked at the risk difference in patients <25 years of age with ADHD, no ADHD, inattentive type ADHD, hyperactive type ADHD, and combined type ADHD who presented with any fracture, a central fracture, an upper limb fracture, a lower limb fracture, an accidental overdose, a burn injury, a drowning incident, a gunshot wounds, suffocation, and a suicide attempt.

**Results:**

Comparison between the no-ADHD cohort and the inattentive, hyperactive/impulsive, combined, and overall ADHD cohorts revealed differences in the majority of outcomes studied. Patients with overall ADHD had significant differences in rates of all outcomes aside from the upper limb fracture. Patients with combined or hyperactive/impulsive ADHD had significant differences in all but drowning, and the inattentive cohort had significance all events.

**Conclusion:**

The stark difference between severe injury presentations in the pediatric emergency department between children with ADHD and without ADHD suggests that providers should consider inquiring whether patients have ADHD to educate them on their risk for severe injuries.

## Introduction

Attention-Deficit/Hyperactive Disorder (ADHD) affects approximately 5% of children globally [1]. This patient population is at risk of several comorbidities as well, as longitudinal studies of pediatric patients with ADHD show that this demographic is at an increased risk for mental health difficulties, social difficulties, and premature mortality when they reach adulthood [2]. One large nationwide cohort study found individuals with ADHD were over twice as likely to die compared to those without ADHD [3]. While these studies detail the comorbid risks of pediatric patients with ADHD, there is currently gap in literature detailing whether children with ADHD are also at increased risk of severe injury and premature mortality while in the developmental age.

Additional studies about severe injuries and mortality among the pediatric population in general suggest that it is indeed reasonable to inquire whether children with ADHD face a greater increased risk for severe injury and childhood mortality. Among these studies are statistics that show that unintentional injuries account for 44% of all injury deaths to children and adolescents [4], one of the most common types of severe injury in the pediatric emergency department is head injury [5], and >12,000 children die from injuries in the United States each year [6]. It is reasonable to suggest that the aforementioned mental health difficulties to which children with ADHD are predisposed can increase a pediatric patient with ADHD's risk for these unintentional injury deaths, severe head injuries, and injury deaths in general. The goal of this study is to perform a review of pediatric emergency department data to illuminate whether there is a difference in the prevalence of certain severe injuries in patients with ADHD compared to patients without ADHD.

## Methods

We performed a retrospective cohort study of patient records contained in the TriNetX database. TriNetX, LLC is compliant with the Health Insurance Portability and Accountability Act (HIPAA), the US federal law which protects the privacy and security of healthcare data, and any additional data privacy regulations applicable to the contributing health care organization [7]. TriNetX is certified to the ISO 27001:2013 standard and maintains an Information Security Management System (ISMS) to ensure the protection of the healthcare data it has access to and to meet the requirements of the HIPAA Security Rule. Any data displayed on the TriNetX Platform in aggregate form, or any patient level data provided in a data set generated by the TriNetX Platform only contains de-identified data as per the de-identification standard defined in Section §164.514(a) of the HIPAA Privacy Rule [7].

We identified all patients under the age of 25 in the TriNetX database who received services in an emergency department between June 12, 2002, and June 12, 2022. Using ICD-10 codes, we identified patients with a diagnosis of ADHD prior to arrival at the ED, including subtype. We compared outcomes between all above patients without ADHD to each specific subtype, as well as to overall patients with the diagnosis of ADHD in their lifetime. The following outcomes were assessed: any fracture (ICD: S_2, 1-9); central fractures involving the skull, face, spine, ribs, sternum, or pelvis (ICD: S02,12,22,32); upper limb fractures involving the shoulder, humerus, forearm, wrist or hand (ICD: S42,52,62); lower limb fractures involving the femur, lower leg, ankle, foot, or toe (ICD: S72,82,92); initial encounter for drowning and nonfatal submersion (ICD: T75.1XXA); suicide attempt (ICD: T14.91); burn (ICD: T30); accidental overdose involving poisoning by drugs, medicaments and biological substances (ICD: T36-50); gunshot wound (GSW) involving handgun discharge, rifle shotgun and larger firearm discharge, other and unspecified firearm discharge, accidental discharge and malfunction from firearms (ICD: Y22-24, W34); suffocation involving foreign body in respiratory tract, asphyxiation, and upper airway obstruction/upper respiratory tract obstruction (ICD: T17, T71, J98.8). The time window for analysis began on the day each patient received services in an emergency department and ended on his or her 25^th^ birthday.

Statistical analysis was performed using the TriNetX software. Comparison between cohorts based on ADHD subtype or absence was performed with a *t*-test. Odds ratios were calculated from outcome incidence within each cohort. To limit confounding variables, we balanced the cohorts on age and sex using propensity score matching with a difference between propensity scores <0.1. Significance for this study was set at *p* < 0.05. As this study contained only deidentified aggregate data, the Colorado Multiple Institutional Review Board (COMIRB) designated it as non-human research not in need of approval.

## Results

**Figure 1:**
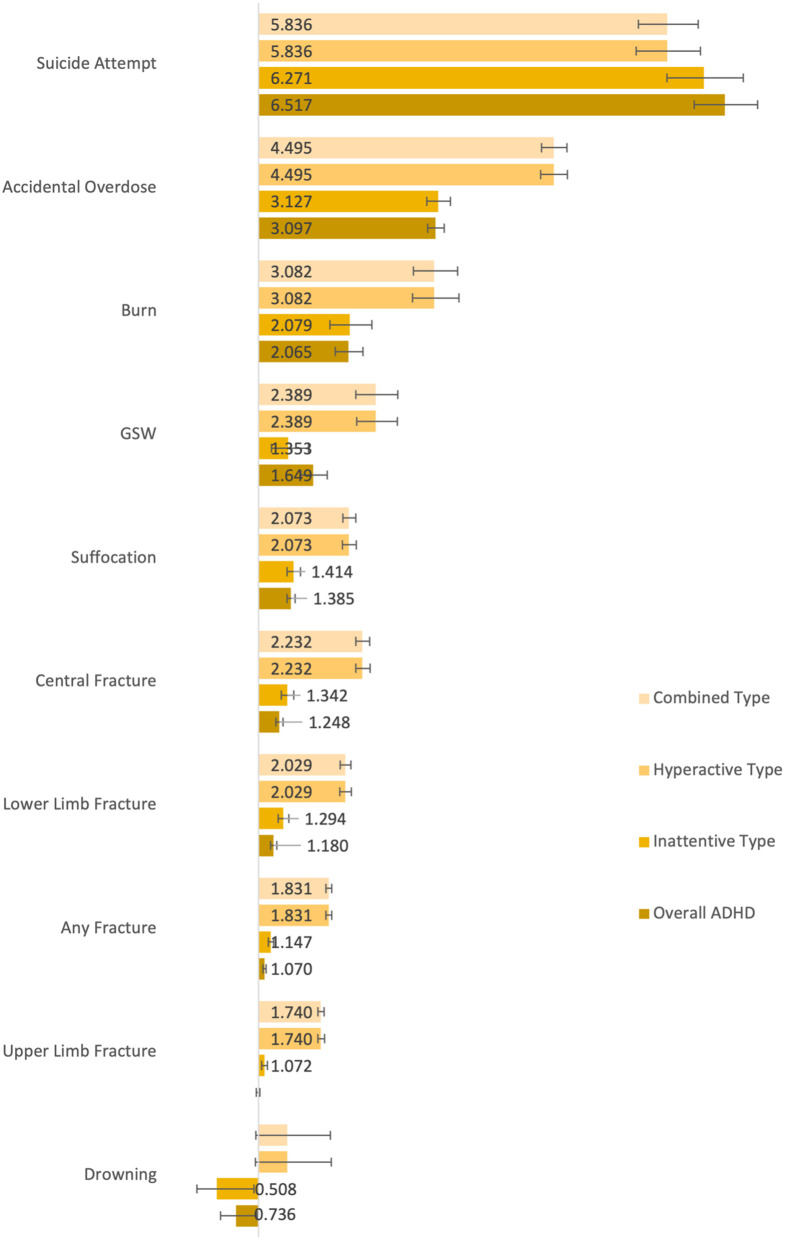
Event odds ratios in comparison to non-ADHD cohort. Bars without numerical labels lack a statistically significant difference.

Once cohorts had been balanced according to age and sex, we identified 301,084 matched pairs of pediatric ED patients where one of the pair had a diagnosis of ADHD prior to ED arrival and the other did not have an ADHD diagnosis in their lifetime. For the subtype analysis, we identified 61,443 youths with predominantly inattentive ADHD, 112,384 youths with predominantly hyperactive/impulsive ADHD, and 160,041 youths with combined type ADHD, each of which were compared to the absence of ADHD cohort. As these subtype cohorts add up to a total of 333,868 patients, it is probable that some patients had more than one ADHD subtype listed in their health record. Comparison of events between the not-ADHD cohort and each of the inattentive, hyperactive/impulsive, combined, and overall ADHD cohorts revealed significant differences in the vast majority of outcomes studied. Patients with overall ADHD had a significant difference in rates of all outcomes aside from the upper limb fracture event. Patients with combined type ADHD had significant differences in every event except drowning, and the inattentive and hyperactive/impulsive cohorts had significance for ten and nine events, respectively. Full results of the subtype analyses are compiled in Table 1. Furthermore, Figure 1 presents a graphical representation of the odds ratio between events. Odds ratio >1 indicate increased likelihood of outcomes, while numbers <1 indicate decreased likelihood.

**Table 1:** Statistics by cohort, including risk, risk difference, odds ratio with CI, and *P* value.

**Event**	**Cohort**	**Event**	**Risk**	**Risk**	**Odds**	**95%**	***P***	**Event**	**Cohort**	**Event**	**Risk**	**Risk**	**Odds**	**95%**	***P***
	**N**	**N**		**diff**.	**ratio**	**CI**	**value**			**N**		**diff**.	**ratio**	**CI**	**value**
**Any fracture**								**Burn**							
No ADHD	301084	30169	10.02%	Control cohort for comparison				No ADHD	301084	915	0.30%	Control cohort for comparison			
Overall ADHD	301084	32059	10.65%	0.63%	1.07	(1.05, 1.09)	< 0.001	Overall ADHD	301084	1883	0.63%	0.32%	2.06	(1.91, 2.24)	<0.001
Inattentive Type	61443	6961	11.33%	1.31%	1.15	(1.12, 1.18)	<0.001	Inattentive type	61443	387	0.63%	0.33%	2.08	(1.85, 2.34)	<0.001
Hyperactive Type	112384	19030	16.93%	6.91%	1.83	(1.8, 1.87)	<0.001	Hyperactive type	112384	1046	0.93%	0.63%	3.08	(2.82, 3.37)	<0.001
Combined Type	160041	23204	14.50%	4.48%	1.83	(1.8, 1.86)	<0.001	Combined type	160041	1314	0.82%	0.52%	3.08	(2.83, 3.35)	<0.001
**Central fracture**								**Drowning**							
No ADHD	301084	6174	2.05%	Control cohort for comparison				No ADHD	301084	106	0.04%	Control cohort for comparison			
Overall ADHD	301084	7669	2.55%	0.50%	1.25	(1.21, 1.29)	<0.001	Overall ADHD	301084	78	0.03%	−0.01%	0.74	(0.55, 0.99)	0.05
Inattentive Type	61443	1679	2.73%	0.68%	1.34	(1.27, 1.42)	<0.001	Inattentive type	61443	11	0.02%	−0.02%	0.51	(0.27, 0.95)	0.048
Hyperactive Type	112384	5016	4.46%	2.41%	2.23	(2.15, 2.32)	<0.001	Hyperactive type	112384	53	0.05%	0.01%	1.34	(0.96, 1.86)	0.545
Combined Type	160041	5741	3.59%	1.54%	2.23	(2.15, 2.31)	<0.001	Combined type	160041	57	0.04%	0.00%	1.34	(0.97, 1.85)	0.67
**Upper limb fracture**								**Gunshot Wound (GSW)**							
No ADHD	301084	20462	6.80%	Control cohort for comparison				No ADHD	301084	685	0.23%	Control cohort for comparison			
Overall ADHD	301084	20336	6.75%	−0.04%	0.99	(0.97, 1.01)	0.518	Overall ADHD	301084	1128	0.37%	0.15%	1.65	(1.5, 1.81)	<0.001
Inattentive Type	61443	4453	7.25%	0.45%	1.07	(1.04, 1.11)	0.029	Inattentive Type	61443	189	0.31%	0.08%	1.35	(1.15, 1.59)	<0.001
Hyperactive Type	112384	12652	11.26%	4.46%	1.74	(1.7, 1.78)	<0.001	Hyperactive Type	160041	867	0.54%	0.31%	2.39	(2.16, 2.64)	<0.001
Combined Type	160041	15366	9.60%	2.81%	1.74	(1.7, 1.78)	<0.001	Combined Type	112384	767	0.68%	0.45%	2.39	(2.15, 2.65)	<0.001
**Lower limb fracture**								**Suffocation**							
No ADHD	301084	8278	2.75%	Control cohort for comparison				No ADHD	301084	5688	1.89%	Control cohort for comparison			
Overall ADHD	301084	9719	3.23%	0.48%	1.18	(1.15, 1.22)	<0.001	Overall ADHD	301084	7820	2.60%	0.71%	1.38	(1.34, 1.43)	<0.001
Inattentive Type	61443	2168	3.53%	0.78%	1.29	(1.23, 1.36)	<0.001	Inattentive type	61443	1629	2.65%	0.76%	1.41	(1.34, 1.5)	<0.001
Hyperactive Type	112384	6096	5.42%	2.67%	2.03	(1.96, 2.1)	<0.001	Hyperactive type	112384	4313	3.84%	1.95%	2.07	(1.99, 2.16)	<0.001
Combined Type	160041	7245	4.53%	1.78%	2.03	(1.97, 2.1)	<0.001	Combined type	160041	5311	3.32%	1.43%	2.07	(2, 2.15)	<0.001
**Accidental overdose**								**Suicide attempt**							
No ADHD	301084	5276	1.75%	Control cohort for comparison				No ADHD	301084	1356	0.45%	Control cohort for comparison			
Overall ADHD	301084	15763	5.24%	3.48%	3.10	(3, 3.2)	<0.001	Overall ADHD	301084	8623	2.86%	2.41%	6.52	(6.15, 6.9)	<0.001
Inattentive type	61443	3246	5.28%	3.53%	3.13	(2.99, 3.27)	<0.001	Inattentive type	61443	1695	2.76%	2.31%	6.27	(5.84, 6.74)	<0.001
Hyperactive type	112384	8342	7.42%	5.67%	4.50	(4.34, 4.66)	<0.001	Hyperactive type	112384	2891	2.57%	2.12%	5.84	(5.47, 6.23)	<0.001
Combined type	160041	10705	6.69%	4.94%	4.50	(4.35, 4.65)	<0.001	Combined type	160041	4700	2.94%	2.49%	5.84	(5.49, 6.2)	0.031

## Discussion

Many of our findings agree with prior research of injury rates in patients with ADHD in comparison to patients without ADHD. Fractures are a common injury type in both the neurotypical (NT) and ADHD populations, and our findings of increased likelihood of certain fracture patterns is in accordance with recent research [8]. Interestingly, the upper limb fracture patterns were the only outcome that did not have a statistically significant difference between the NT and ADHD cohorts. We hypothesize this finding may be due to mechanisms of injury involved in different fracture patterns; while central fracture patterns are often found in motor vehicle accidents, upper limb fractures are common in sports and accidental falls, which are not known to be more common in patients with ADHD [9]. Skull and face fractures, which are included within central fractures, are a leading cause of disability in pediatric emergency department patients, and have become even more common since the start of the COVID-19 pandemic [5]. Given the increased propensity for central fractures in patients with ADHD, our findings suggest further investigation into injury prevention could benefit this vulnerable patient population.

Our subtype analysis offered novel insight into the relationship between the injury mechanisms and ADHD symptomatology. Specifically, patients with combined type ADHD had higher likelihood of all but one outcomes compared to the absence of ADHD population. As a diagnosis of combined type necessitates meeting the criteria for both inattentive and hyperactive types, these patients experience a greater number of ADHD symptoms, and potentially a more intense symptomatology overall [10]. Furthermore, the co-occurrence of hyperactivity/impulsivity and inattention could have an additive effect on many of the outcomes of bodily harm measured in our study. Events such as drowning, burns, gunshot wounds, and accidental overdose involve an element of both situational and personal determinants; the hyperactive or impulsive symptoms may put a youth with ADHD at greater risk for a potentially dangerous situation, and the inattentive symptoms may contribute to the pretermission of proper safety precautions, leading to the measured injury outcome.

Patients with a diagnosis of hyperactive/impulsive type ADHD had the greatest significant increase in risk for 9 of the 10 outcomes, with drowning being the exception. We hypothesize that the decreased proprioception and loss of self-inhibition present in hyperactive/impulsive type ADHD contributes to the increased occurrence of injury, particularly fracture, found in this specific subtype population. Neither the hyperactive/impulsive type nor the combined type had significant differences in occurrence of drowning events compared to non-ADHD control (Hyperactive: OR = 1.34, *p* = 0.055; Combined: OR = 1.34, *p* = 0.67). However, the overall incidence of drowning event in our population was low compared to other outcomes (*N* = 78), which could contribute to the lack of statistical significance. Similarly, the inattentive type cohort was the smallest of all populations in our study (*N* = 61,443). For the events with significant increased likelihood, the difference in rates compared to control was much smaller than the other subtypes of ADHD. Interestingly, inattentive youths were less likely to present with drowning compared to the not-ADHD cohort (OR = 0.82, *p* < 0.001), which again may be accounted for by differences in behavior; inattentive type ADHD is characterized by daydreaming, wandering, and disengagement [10]. We hypothesize that disengagement specifically may explain some of the difference in injuries seen.

Our findings of suicide attempt likelihood are particularly relevant given the increasing number of attempted suicides in the general pediatric population, as well as the growing proportion of emergency department visits relating to behavioral health [11, 12]. Patients without ADHD were <1/6^th^ as likely to attempt suicide compared to the overall ADHD cohort, which is in agreement with prior research on the association between suicidality and ADHD [13]. Within the population diagnosed with ADHD, all subtypes were slightly less likely to attempt suicide than the not-ADHD cohort (Hyperactive: OR = 5.84, *p* < 0.001; Inattentive: OR = 6.27, *p* < 0.001; Combined: OR = 5.84, *p* = 0.031). As hypothesized above, it is possible that the presence of both inattentive and hyperactive/impulsive symptoms increases the risk factors for suicide in youths, such as social and familial conflict [14]. Youths with ADHD are known to have trouble connecting socially with their peers and are known to have a higher risk of mental health struggles such as depression [1], which could contribute to our finding of differences in likelihood of suicide attempts between patients with and without ADHD.

Emergency department providers are severely overworked, and it is unlikely that ADHD would be at the forefront of a provider's mind while treating a child with a gunshot wound or accidental overdose. ED providers should not be expected to diagnose a child with ADHD; however, our findings suggest it may improve patient care to ensure that youths presenting to the emergency department are asked about previous ADHD diagnosis. Prior research has shown that medication treatment leads to decreased overall mortality in patients with ADHD, whereas delayed treatment leads to a further increased mortality [15]. Patients with ADHD have also been found to be significantly more likely to require multiple visits to an emergency department [16]. Given the large differences in event risk between patients with and without ADHD, long term patient outcomes may be improved if ED providers specifically ask about ADHD and suggest patients return to their psychiatrist or primary care provider to ensure appropriate medication management.

As with all studies, the generalizability of our findings is limited by the source of our data. The deidentified nature of the data makes it extremely difficult to control for certain social determinants of health relevant in pediatric emergency care, such as geographic location and family status. Additionally, a small portion of our patients with ADHD may have had diagnoses for multiple subtypes and would be present in the analysis for each of the subtypes. Similarly, a patient with both drowning and a fracture would be present in two analyses. However, the impact is likely small as the TriNetX software is restricted to two group analysis, so direct comparison between subtypes was not performed.

Our findings should not end the investigation into severe injury within subtypes of ADHD, as our data did not examine two meaningful variables: medications and comorbidities. These variables were not separated by event analysis as there is currently no established research detailing differences of medication management and comorbidity rates by subtype of ADHD. Rather than attempt to address two novel areas of investigation, we expanded the field of injury research in ADHD, which had not separated analysis by subtype until our research.

## Conclusion

Our findings reinforce that of prior research and provide novel insight into the increased overall occurrence of injury and mortality in youths with ADHD. As issues such as gunshot wounds and suicidal ideation become increasingly researched by the pediatric emergency care community, it is important that providers are aware of the medical, social, and behavioral factors that place a child at increased risk for these events. It is our hope that our findings better enable the ED provider to counsel, inform, and refer youths with ADHD to their psychiatrist or primary care provider, and mitigate the increased overall morbidity and mortality in this population.

## Data availability statement

The original contributions presented in the study are included in the article/supplementary material, further inquiries can be directed to the corresponding author.

## Author contributions

All authors listed have made a substantial, direct, and intellectual contribution to the work and approved it for publication.

## Conflict of interest

The authors declare that the research was conducted in the absence of any commercial or financial relationships that could be construed as a potential conflict of interest.

## Publisher's note

All claims expressed in this article are solely those of the authors and do not necessarily represent those of their affiliated organizations, or those of the publisher, the editors and the reviewers. Any product that may be evaluated in this article, or claim that may be made by its manufacturer, is not guaranteed or endorsed by the publisher.
